# Reasons for Hospitalizations and Emergency Department Visits Among Patients with Essential Tremor

**DOI:** 10.5334/tohm.934

**Published:** 2024-09-23

**Authors:** Susanna Howard, Ellie Gabriel, Ritesh Karsalia, Dominick Macaluso, Jesse Y. Hsu, Liming Qiu, Neil R. Malhotra, Iahn Cajigas, Whitley Aamodt, John Farrar

**Affiliations:** 1 Department of Neurosurgery, University of Pennsylvania, Philadelphia, PA, USA; 2 University of Pennsylvania Perelman School of Medicine, Philadelphia, PA, USA; 3 Department of Biostatistics, Epidemiology and Informatics, Perelman School of Medicine, University of Pennsylvania, Philadelphia, PA, USA; 4 Department of Neurology, University of Pennsylvania, Philadelphia, PA, USA

**Keywords:** essential tremor, healthcare resource utilization

## Abstract

**Background:**

Prior studies suggest that patients with essential tremor (ET) have increased rates of healthcare utilization, but the reason for this increased use is unknown. The objective of this study was to evaluate the reasons for healthcare use among ET patients.

**Methods:**

This was a retrospective cross-sectional study of ET patients with an admission or emergency department (ED) visit at a tertiary health system from 2018–2023. Patients were matched on an encounter level with control patients based on propensity scores incorporating age, sex, race, and co-morbid conditions. The primary outcome was the odds of an encounter for each diagnostic category comparing ET patients with matched controls.

**Results:**

Only inpatient admissions for neurologic diagnoses were more likely for ET compared to control patients (odds ratio (OR) 3.73, 95% confidence interval (CI) 2.54 – 5.49, p < 0.001). Once admissions related to the surgical treatment of tremor were excluded, admissions for neurologic diagnoses were equally likely among ET and control patients (OR 0.96, 95% CI 0.59 – 1.57, p = 0.88).

**Discussion:**

Surgical treatment of tremor appears to be a key driver of healthcare use among ET patients. Future investigations should examine the pattern of healthcare use of ET patients before and after surgery.

**Highlights:**

Prior studies have shown increased healthcare use among essential tremor (ET) patients. The objective of this study was to evaluate the reasons for healthcare use among ET patients compared to matched control patients. Surgical treatment of tremor was found to be a key driver of healthcare use among ET patients.

## Introduction

Essential tremor (ET) is the most common movement disorder worldwide [1]. While ET has been associated with detrimental impacts on quality of life, the symptoms of tremor itself do not typically necessitate an ED visit or inpatient admission [2,3,4,5,6]. However, recent publications have suggested that patients with ET have higher healthcare utilization compared to matched control patients without ET [7,8]. In an analysis of administrative claims data from 2017–2019, patients with ET had higher admission rates and emergency department (ED) visits compared to control patients without ET matched based on age, gender, payer type, and ZIP code (inpatient admission: 21% with ET vs. 16.5% without ET, p < 0.0001; at least one ED visit 30.2% with ET vs. 25.0% without ET, p < 0.0001) [8]. Although there was greater healthcare utilization among patients with ET, the reasons for these ED visits and hospitalizations were not explored. To expand on these findings, we conducted a retrospective study using electronic health record (EHR) data from the University of Pennsylvania Health System (UPHS) to compare the reasons for inpatient admissions and ED visits among patients with ET versus randomly selected control patients without ET matched based on demographic and clinical characteristics.

## Methods

We conducted a retrospective cross-sectional study of patients with International Classification of Diseases, 10^th^ Revision (ICD) codes for ET with inpatient admissions and/or ED visits within UPHS from January 1^st^, 2018, to January 1^st^, 2023. Herein, encounter will be the term used to refer to either inpatient admission or ED visits.

### Patient Population

The EHR was queried for inpatient admissions and ED visits from January 1^st^, 2018, to January 1^st^, 2023. Encounters associated with patients less than 18 years of age were excluded, as well as encounters missing an associated diagnostic group. Encounters associated with patients who had Parkinson’s disease were excluded to minimize misclassification. Encounters were classified in the ET group if the ICD-10 code G25.0 was present within the associated patient’s medical history or problem list at least once during the study period [9]. Control encounters were associated with patients without an ET diagnosis. Analysis was conducted at the encounter level; therefore, a single patient could be associated with multiple encounters in the sample. If a single patient had both inpatient admissions and ED visits during the study period, both encounter types were included and analyzed separately.

### Covariates and Matching

The following covariates were considered: age, sex, race, and medical conditions used in the Charlson Comorbidity Index (CCI) [10]. The ICD-10 codes used to identify the CCI medical conditions were based on previously published coding tables [11]. A propensity score for the likelihood of having an ET diagnosis was estimated using these covariates, separated by inpatient and ED visits. These propensity scores were used to create matched samples such that the ET encounters and the matched non-ET encounters have similar covariate distributions. Eighteen out of the 19 CCI conditions were used in the propensity score for inpatient admissions—complicated diabetes was omitted due to collinearity with diabetes without chronic complications. Seventeen CCI conditions were used in the propensity score for ED visits—complicated diabetes and acquired immune deficiency syndrome were omitted due to collinearity with diabetes without chronic complications and human immunodeficiency virus, respectively. One-to-one matching between ET encounters and control encounters was performed based on the logit of the propensity score. Matching without replacement was initiated with the highest propensity scores and proceeded in a descending manner. Matches were required to have an absolute difference in the logit of propensity scores that was no greater than 0.2 of the standard deviation of the logit of the propensity scores. Standardized mean differences between ET encounters and encounters of control patients >0.1 were interpreted as covariate imbalance [12].

### Outcome Measures

The primary outcome was the presence of a particular diagnostic group associated with an encounter (inpatient admission or ED visit). The diagnostic group is based on the ICD code of the principal diagnosis associated with each encounter. The list of diagnostic groups with the corresponding ICD codes is provided in Supplementary Table 1.

### Statistical Analysis

Analyses were performed separately for inpatient admissions and ED visits. Baseline and demographic characteristics were summarized using descriptive statistics. Among the matched pairs, a logistic regression model was used to determine the odds of an encounter associated with each diagnostic group among patients with ET compared to control patients. To account for the correlation between groups induced by matching, cluster-robust standard errors at the matched pair level were used. Statistical significance was defined as a *p*-value of less than 0.002 after a Bonferroni correction considering 22 possible outcomes. Stata version 18 (StataCorp, College Station, TX, USA) was used for statistical analysis.

## Results

### Inpatient Admissions

Baseline demographics and co-morbidities of the unadjusted and matched samples of inpatient admissions are shown in Table 1. The distribution of propensity scores before and after matching is shown in Supplementary Figure 1. Among the 888 ET inpatient admissions, 600 unique patients were represented. Of these 600 unique patients with ET, 192 (13.0%) patients also had at least one ED encounter during the study period. Among the 888 control inpatient admissions, 877 unique patients were represented. Of these 877 unique patients without ET, 3 (0.2%) patients also had at least one ED encounter included during the study period.

**Table 1:** **Comparison of demographic characteristics and Charlson Comorbidity Index (CCI) co-morbidities between inpatient admissions among the unadjusted and matched samples**. Continuous variables are reported as medians with interquartile range. *Four admissions in the unadjusted sample were missing data regarding race and were not included in the matched sample. Abbreviations: AIDS, acquired immune deficiency syndrome; HIV, human immunodeficiency virus.


	UNADJUSTED			PROPENSITY-SCORE MATCHED		

COVARIATE	CONTROL ADMISSIONS (TOTAL N = 157,594) n (%)	ET ADMISSIONS (TOTAL N = 893) n (%)	STANDARDIZED MEAN DIFFERENCE	CONTROL ADMISSIONS (TOTAL N = 888) n (%)	ET ADMISSIONS (TOTAL N = 888) n (%)	STANDARDIZED MEAN DIFFERENCE

Age, years	63.7 (47.6 – 74.3)	73.1 (67.5 – 79.9)	0.78	73.2 (65.3 – 82.3)	73.1 (67.4 – 79.9)	0.02

Female	84,891 (53.9)	474 (53.1)	–0.02	443 (49.9)	471 (53.0)	0.06

Race*			–0.52			0.05

White	97,018 (63.2)	779 (87.6)		795 (89.5)	778 (87.6)	

Black	42,130 (27.4)	87 (9.8)		80 (9.0)	87 (9.8)	

Asian	6,077 (4.0)	9 (0.9)		4 (0.5)	9 (1.0)	

American Indian or Alaskan Native	328 (0.2)	0		0	0	

Native Hawaiian or Pacific Islander	309 (0.2)	0		0	0	

Other or Unknown	7,702 (5.0)	14 (1.6)		9 (1.0)	14 (1.6)	

CCI co-morbidities						

Myocardial infarction	15,274 (9.7)	88 (9.9)	0.00	81 (9.1)	88 (9.9)	0.03

Congestive heart failure	34,646 (22.0)	195 (21.8)	–0.01	172 (19.4)	195 (22.0)	0.06

Peripheral vascular disease	24,549 (15.6)	203 (22.7)	0.18	171 (29.3)	202 (22.7)	0.09

Cerebrovascular disease	28,600 (18.1)	205 (23.0)	0.12	203 (22.9)	204 (23.0)	0.00

Dementia	8,226 (5.2)	83 (9.3)	0.16	79 (8.9)	83 (9.3)	0.02

Chronic pulmonary disease	38,251 (24.3)	285 (31.9)	0.17	244 (27.5)	284 (32.0)	0.10

Rheumatic disease	6,604 (4.2)	64 (7.2)	0.13	62 (7.0)	63 (7.1)	0.01

Peptic ulcer disease	4,582 (2.9)	34 (3.8)	0.05	33 (3.7)	34 (3.8)	0.01

Liver disease, mild	12,695 (8.1)	97 (10.9)	0.09	101 (11.4)	97 (10.9)	–0.02

Diabetes without complications	44,183 (28.0)	317 (35.5)	0.16	315 (35.5)	315 (35.5)	0.00

Renal disease, mild to moderate	27,649 (17.5)	228 (25.5)	0.19	200 (22.5)	227 (25.6)	0.07

Diabetes with complications	44,183 (28.0)	317 (35.5)	0.16	315 (35.5)	315 (35.5)	0.00

Hemiplegia or paraplegia	2,251 (1.4)	24 (2.7)	0.09	27 (3.0)	23 (2.6)	–0.03

Any malignancy	42,683 (27.1)	274 (30.7)	0.07	280 (31.5)	273 (30.7)	–0.02

Liver disease, moderate to severe	2,711 (1.7)	11 (1.2)	–0.04	11 (1.2)	11 (1.2)	0.00

Renal disease, severe	10,785 (6.8)	41 (4.6)	–0.10	43 (4.8)	41 (4.6)	–0.01

HIV infection, no AIDS	1,971 (1.3)	7 (0.8)	–0.05	14 (1.6)	7 (0.8)	–0.08

Metastatic solid tumor	7,144 (4.5)	32 (3.6)	–0.06	29 (3.3)	31 (3.5)	0.01

AIDS	681 (0.4)	3 (0.3)	–0.02	8 (0.9)	3 (0.3)	–0.09


**Figure 1:**
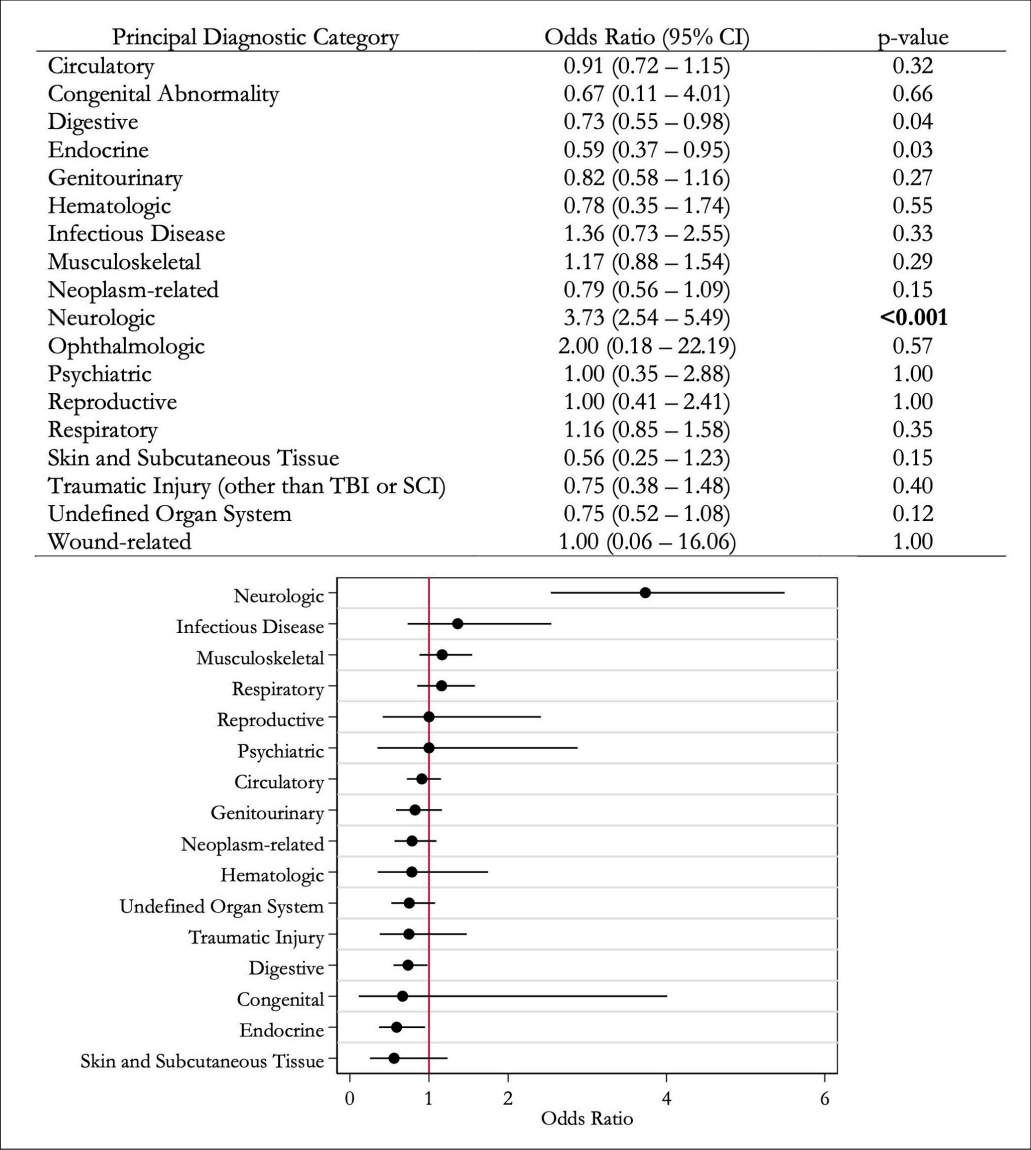
**Odds of inpatient admission associated with each diagnostic category among admissions of patients with essential tremor (ET) (n = 888) compared to matched admissions among control patients without ET (n = 888)**. Odds of ophthalmologic and wound-related admissions were omitted from the plot due to the CI widths. Due to multiple comparisons, statistical significance was defined as a *p*-value of less than 0.002. Abbreviations: CI confidence interval; SCI spinal cord injury; TBI traumatic brain injury.

Supplementary Table 2 shows the frequency of inpatient admissions associated with each diagnostic category. Among patients with ET, the highest number of inpatient admissions were circulatory-related (163/888, 18.4%). Figure 1 shows the odds of inpatient admission associated with each diagnostic category among admissions of patients with ET compared to matched control patients. Only inpatient admissions associated with a neurologic diagnosis were significantly more likely among patients with ET (OR 3.73, 95% CI 2.54 – 5.49, p < 0.001), and the majority of these admissions (92/124, 74.2%) were associated with an ET diagnosis and related to the surgical treatment of tremor (77 admissions for deep brain stimulation (DBS) implantation, 7 admissions for magnetic resonance-guided focused ultrasound (MRgFUS) thalamotomy, and 8 admissions related to a surgical complication). Neurologic admissions unrelated to surgery for tremor among the ET group included: undefined neurologic diagnosis including altered mental status (11/124), spine pathologies (5/124), brain tumors (5/124), seizures (3/124), Bell’s palsy (2/124), and trigeminal neuralgia (1/124). When the admissions related to the surgical treatment of tremor were excluded, admissions related to a neurologic diagnosis among patients with ET were no longer significantly higher (OR 0.96, 95% CI 0.59 – 1.57, p = 0.88). When the sample was restricted to patients with a single admission (Supplementary Table 3), inpatient admissions associated with a neurologic diagnosis were significantly more likely among patients with ET (OR 6.05, 95% CI 3.95 – 9.26, p < 0.001). When admissions related to surgery for tremor (n = 65) were excluded from this restricted sample, admissions related to neurologic diagnosis were no longer significantly more likely among patients with ET (OR 1.31, 95% CI 0.73 – 2.37, p = 0.37).

### ED Visits

Baseline demographics and co-morbidities of the unadjusted and matched samples of ED visits are shown in Table 2. Supplementary Figure 1 shows the distribution of propensity scores before and after matching. Among the 1,114 ET ED visits, 676 unique patients were represented. Among the 1,114 control ED visits, 1088 unique patients were represented.

**Table 2:** **Comparison of demographic characteristics and Charlson Comorbidity Index (CCI) co-morbidities between emergency department (ED) visits among the unadjusted and matched samples**. Continuous variables are reported as medians with interquartile range. *Two ED visits in the unadjusted sample were missing data regarding race and were not included in the matched sample. Abbreviations: AIDS acquired immune deficiency syndrome; HIV human immunodeficiency virus.


	UNADJUSTED			PROPENSITY-SCORE MATCHED		

COVARIATE	CONTROL ED VISITS(TOTAL N = 335,417) n (%)	ET ED VISITS(TOTAL N = 1,116) n (%)	STANDARDIZED MEAN DIFFERENCE	CONTROL ED VISITS(TOTAL N = 1,114) n (%)	ET ED VISITS(TOTAL N = 1,114) n (%)	STANDARDIZED MEAN DIFFERENCE

Age, years	34.2 (25.1 – 52.8)	69.5 (57.9 – 78.3)	1.46	70.3 (58.6 – 81.7)	69.6 (57.9 – 78.3)	–0.10

Female	181,165 (54.0)	643 (57.6)	0.07	617 (55.4)	643 (57.7)	0.05

Race*			–0.52			0.07

White	146,843 (46.8)	826 (74.1)		870 (78.1)	826 (74.1)	

Black	117,255 (37.4)	220 (19.7)		189 (17.0)	220 (19.7)	

Asian	20,372 (6.5)	24 (2.2)		17 (1.5)	24 (2.2)	

American Indian or Alaskan Native	944 (0.3)	0		0	0	

Native Hawaiian or Pacific Islander	937 (0.3)	0		0	0	

Other or Unknown	27,576 (8.8)	44 (3.9)		38 (3.4)	44 (3.9)	

CCI co-morbidities						

Myocardial infarction	5,954 (1.8)	53 (4.7)	0.16	73 (6.6)	53 (4.8)	–0.10

Congestive heart failure	10,309 (3.1)	161 (14.4)	0.40	166 (14.9)	161 (14.5)	–0.02

Peripheral vascular disease	7,742 (2.3)	183 (16.4)	0.49	159 (14.3)	183 (16.4)	0.08

Cerebrovascular disease	14,170 (4.2)	220 (19.7)	0.48	184 (16.5)	220 (19.7)	0.20

Dementia	5,505 (1.6)	101 (9.1)	0.33	95 (8.5)	101 (9.1)	0.02

Chronic pulmonary disease	58,458 (17.4)	390 (34.9)	0.39	359 (32.2)	389 (34.9)	0.06

Rheumatic disease	4,695 (1.4)	72 (6.5)	0.26	55 (4.9)	77 (6.5)	0.08

Peptic ulcer disease	3,388 (1.0)	44 (3.9)	0.19	30 (2.7)	44 (3.9)	0.08

Liver disease, mild	8,492 (2.5)	94 (8.4)	0.26	90 (8.1)	94 (8.4)	0.02

Diabetes without complications	32,597 (9.7)	347 (31.1)	0.55	308 (27.6)	347 (31.1)	0.09

Renal disease, mild to moderate	8,575 (2.6)	205 (18.4)	0.53	188 (16.9)	205 (18.4)	0.05

Diabetes with complications	32,597 (9.7)	347 (31.1)	0.55	308 (27.6)	347 (31.1)	0.09

Hemiplegia or paraplegia	1,228 (0.4)	13 (1.2)	0.09	16 (1.4)	13 (1.2)	–0.03

Any malignancy	17,693 (5.3)	248 (22.2)	0.50	231 (20.7)	248 (22.3)	0.05

Liver disease, moderate to severe	843 (0.3)	4 (0.4)	0.02	6 (0.5)	4 (0.4)	–0.03

Renal disease, severe	3,151 (0.9)	43 (3.9)	0.19	49 (4.4)	43 (3.9)	–0.03

HIV infection, no AIDS	2,784 (0.8)	19 (1.7)	0.08	14 (1.3)	19 (1.7)	0.04

Metastatic solid tumor	1,940 (0.6)	16 (1.4)	0.08	13 (1.2)	16 (1.4)	0.03

AIDS	384 (0.1)	0	–0.05	0	0	0.00


**Figure 2:**
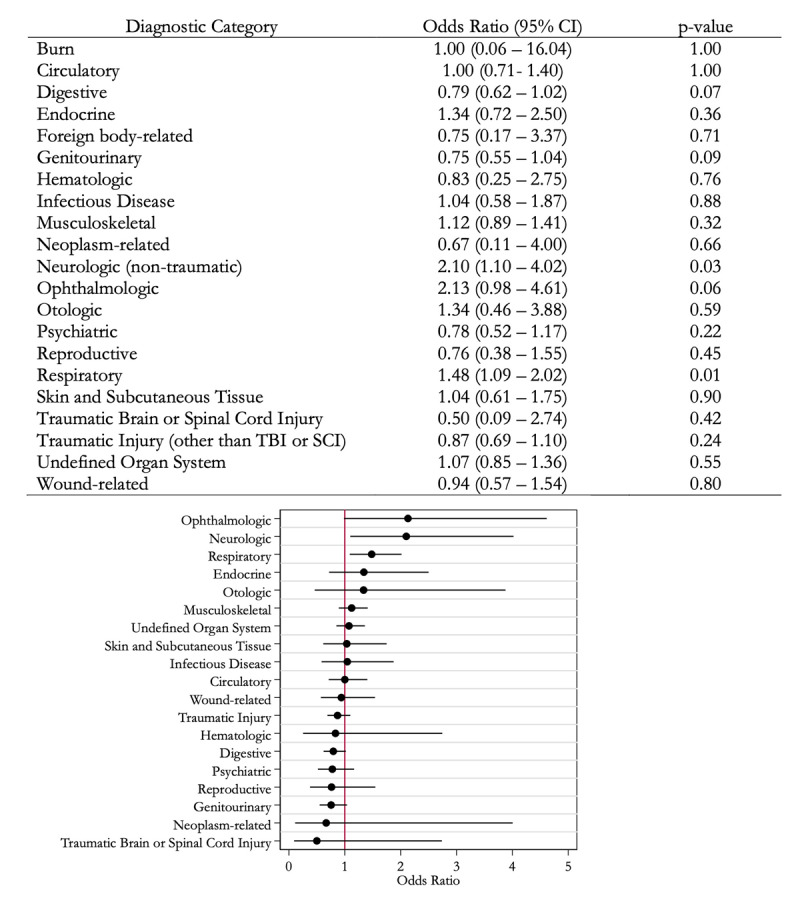
**Odds of emergency department (ED) visits associated with each diagnostic category among ED visits of patients with essential tremor (ET) (n = 1,114) compared to matched control ED visits among patients without ET (n = 1,114)**. Odds of burn-related visits were omitted from the plot due to the CI width. Due to multiple comparisons, statistical significance was defined as a *p*-value of less than 0.002. Abbreviations: CI confidence interval; SCI spinal cord injury; TBI traumatic brain injury.

Supplementary Table 4 shows the frequency of ED visits associated with each diagnostic category. In the ET group, the highest number of ED visits were musculoskeletal-related (184/1,114, 16.5%). Figure 2 shows the odds of ED visits associated with each diagnostic category among patients with ET compared to matched control patients without ET. There were no differences in the odds of visits from any diagnostic categories between patients with ET and control patients that met the threshold of statistical significance (*p*-value < 0.002).

## Discussion

Our study shows that the reasons for ED visits and inpatient admissions are largely similar between patients with ET and matched patients without ET. Admissions for neurologic diagnoses were only significantly more likely among patients with ET when admissions related to surgery for tremor were included. However, when these surgical admissions were removed, there was no substantial difference in total admissions, diagnostic conditions, or ED visits, thus supporting the concept that ET-related surgeries may be driving the increased healthcare utilization previously reported in this population.

Importantly, the two prior studies showing patients with ET had higher healthcare utilization and expenditures compared to matched control patients did not provide results explaining the reasons for healthcare use, and surgical admissions among patients with ET were not excluded in either study [7,8]. In the first analysis of Medicare beneficiaries with ET, the number of patients who received surgery for ET was not reported [7]. In the second analysis of Aetna’s administrative claims from 2017–2019, Dai et al divided ET patients into three categories: 1) an untreated group with no evidence of claims for ET-specific treatment; 2) a pharmacotherapy group with prescriptions for medications used to treat ET; 3) an invasive group with evidence of surgical treatment for ET [8]. Among the ET patients, patients undergoing invasive therapy for ET (DBS or thalamotomy) had significantly higher rates of ED visits and inpatient admission rates compared to patients with ET receiving medical therapy or no treatment in the one-year post-index period. Admissions for surgery itself could be the reason for the increased admissions in the invasive group. The increased rate of ED visits could also be explained by surgery. Despite the overall favorable safety profile of DBS, complications could require an ED visit. Among 215 patients with a history of DBS implantation, a retrospective chart review found that 13% of patients had an ED visit for a DBS-related issue [13]. In addition, the increased rate of inpatient admissions and ED visits in the pharmacotherapy group could also be associated with the treatment itself. The discontinuation rates of medications for ET are high [14,15]. A nationwide claims analysis found that 40% of patients discontinued medication for ET within two years of initiation [14]. Medication discontinuation is most commonly attributed to a lack of efficacy and side effects [14,15]. If the side effects from tremor medications are severe (e.g., symptomatic bradycardia from beta-blockers), treatment complications could prompt presentation to an ED. In the aforementioned analysis of Aetna’s administrative claims, the categorization of ET patients did not match by or adjust for age or co-morbid conditions, therefore the conclusions made regarding the reasons for healthcare use are limited [8]. Dai et al found that patients with ET in the untreated group had similar rates of healthcare use to patients without evidence of an ET diagnosis.

The need for pharmacotherapy and/or surgical treatment is likely a surrogate marker of ET severity. Given that surgical treatment for ET appears to be a key driver of inpatient admissions among patients with ET, these surgeries must be cost-effective. Previously published decision analysis models have identified MRgFUS thalamotomy as a more cost-effective procedural option for treating ET compared to DBS [16,17]. Unlike DBS, MRgFUS thalamotomy is typically an outpatient procedure and does not require inpatient admission. However, certain patients are not ideal candidates for MRgFUS thalamotomy due to bilateral symptoms of ET or low skull density ratio; these characteristics can make DBS a more optimal approach [18,19,20]. Efforts to increase the cost-efficacy of DBS are ongoing. A recent single-center retrospective analysis demonstrated that outpatient DBS surgery had a comparable safety profile with inpatient DBS surgery [21]. Future studies should also investigate the pattern of healthcare use of ET patients before and after surgery. If the rate of healthcare use decreases following surgery, it could suggest that tremor reduction and quality-of-life improvements after surgery lead to decreased non-ET-related healthcare needs [22,23].

In our study, there were substantial differences between the ET and control encounters in the baseline crude unmatched sample. Patients with ET were older among both the ED visits and inpatient admissions compared to the control patients. This observation fits with the knowledge that the prevalence of ET markedly increases with age [1]. Consistent with older age, the prevalence of co-morbidities was higher among ET patients compared to control patients in the unmatched sample (8 of 19 CCI conditions among inpatient admissions, 14 of 19 CCI conditions among ED visits). The increase in number of chronic conditions with age is a well-proven epidemiologic observation [24,25]. For example, an analysis of approximately 31 million Medicare fee-for-service beneficiaries found that 50% of people under the age of 65 years had two or more chronic conditions compared to 81.5% of people aged 85 years or older [24]. Prior studies have also found an increased rate of co-morbidities in ET patients compared to patients without ET even when controlling for age [8,26,27]. Despite matching by age, Dai et al found that patients with ET had a higher mean number of comorbid conditions compared to patients without ET (5.26 (standard deviation (SD) 3.21) vs. 4.03 (SD 3.27), p < 0.0001) [8]. If the overall burden of illness is higher among ET patients compared to the general population, this could certainly lead to increased healthcare use and costs. The increased rate of comorbid conditions among ET patients could also reflect differences in health-seeking behavior rather than true health status. Patients with greater health-seeking behavior may be more likely to receive a diagnosis of ET as well as other medical diagnoses. However, a prior study using a model that adjusted for total number of healthcare visits as a proxy for health-seeking behavior still found that certain disorders (e.g., depression, alcohol abuse, pulmonary disease, etc.) were more likely among ET patients compared to matched control patients [27]. Providers providing specialized care for patients with ET should be aware of this trend and ensure co-morbid conditions are adequately addressed.

In interpreting our findings, we need to consider the limitations, many of which are common to all retrospective observational cohort studies. Matching based on age, sex, race, and CCI co-morbidities was used to select controls to limit potential confounding variables outside of the exposure of interest—ET. The logistic regression model used to determine the odds of an encounter associated with each diagnostic category accounted for similarities introduced via propensity-score matching with cluster-robust standard errors. However, this model does not account for possible correlation between multiple encounters of the same patient. Each encounter was treated as a unique observation given the primary objective was to assess the etiology of healthcare use, however, this could cause a single patient with multiple encounters to contribute disproportionately to the results. Another limitation of this study was that encounters in certain diagnostic categories occurred rarely (<10) leading to odds ratios with wide confidence intervals. A larger sample size would be necessary to have the statistical power to definitively conclude there was no difference in the reasons for admissions and ED visits between the ET and control groups. Due to the higher prevalence of comorbid conditions among ET populations, some experts have hypothesized that ET is a disease complex [8,26,27]. Therefore, by matching based on comorbid conditions, our study design could obscure differences in healthcare use driven by conditions associated with ET. While we acknowledge this potential limitation, the CCI conditions used in the propensity score are not an exhaustive list of comorbidities, and conditions such as depression and anxiety previously associated with ET are not included [26]. Due to prior studies showing that ET patients had a higher number of inpatient admissions and ED visits compared to controls, our study focused on these types of healthcare encounters specifically [7,8]. Our results provide important insights into the reasons for healthcare use in these settings but cannot be generalized to the outpatient setting. Examining reasons for outpatient healthcare use among ET patients compared to patients without ET could be the focus of a future investigation. The setting of a tertiary health system may also limit the generalizability of our findings. Less than 3% of all patients with ET undergo invasive treatments, however, the proportion of ET patients receiving surgery may be higher in a health system with greater neurosurgical capabilities like UPHS [8,14].

In conclusion, patients with ET were older with a greater number of co-morbid conditions compared to patients without ET in our crude unmatched sample. When surgical admissions for tremor are excluded, and patients are matched based on a propensity score using age, sex, race, and co-morbid conditions, the reasons for healthcare use among ET patients were similar to matched control patients. In this tertiary medical system, surgical treatment of ET appears to be a key driver of healthcare use among the ET population. Surgical treatment has been shown to improve the quality of life of patients with ET, however, future studies should examine whether these functional improvements translate to decreased healthcare use post-operatively [22,28,29].

## Additional File

The additional file for this article can be found as follows:

10.5334/tohm.934.s1 Supplementary File 1. Figure 1 and Tables 1 to 4.

## Financial Disclosures

In the past three years, Dr. Farrar has received compensation for serving on two NIH data safety monitoring boards and advisory boards or consulting on clinical trial methods from Vertex Pharma and EicOsis Pharma. The other authors have no financial disclosures.
