# Supplementary material for: Reasons for Hospitalizations and Emergency Department Visits Among Patients with Essential Tremor
Source: Tremor Other Hyperkinet Mov (N Y). 2024 Sep 23;14:47. doi: 10.5334/tohm.934 (PMC11428660; doi:10.5334/tohm.934)

Supplementary Figure 1. Propensity score distribution for unadjusted and matched samples for A) inpatient admissions and B) emergency department (ED) visits. Abbreviations: ET, essential tremor.

A)

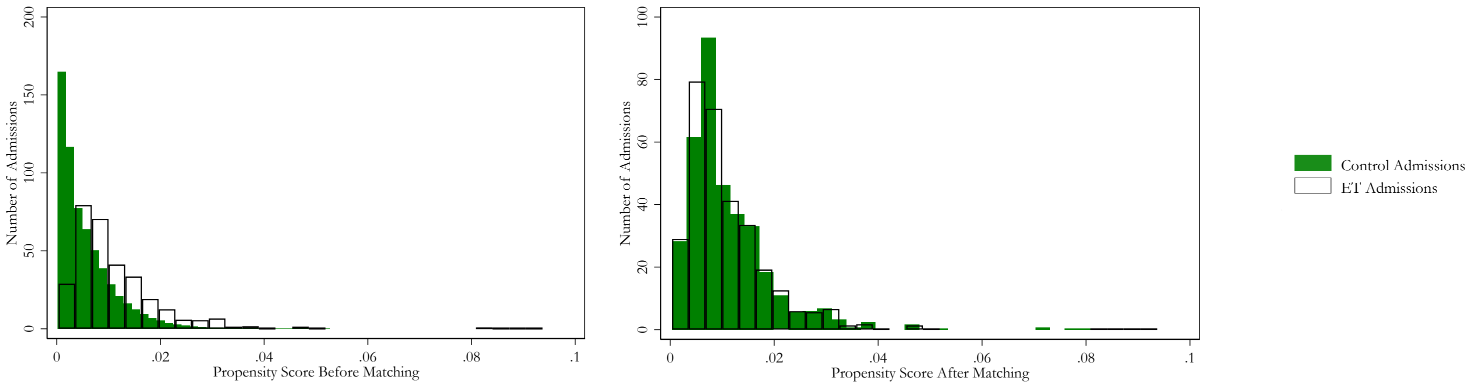

B)

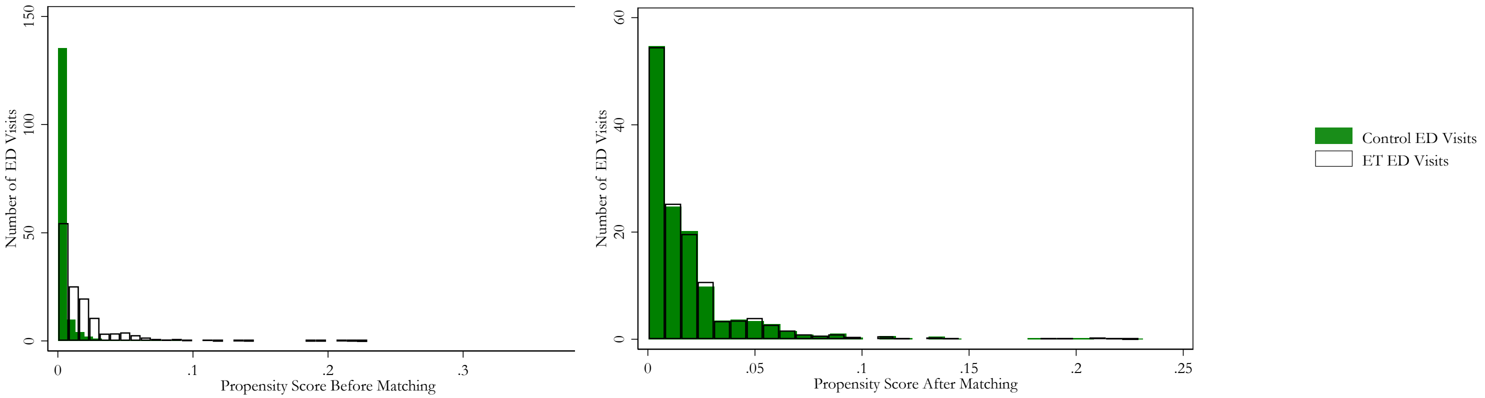

Supplement: Supplementary File 1. — Figure 1 and Tables 1 to 4. [file tohm-14-1-934-s1.zip › tohm-934_howard-s1/Supplementary Figure 1.pdf]
