# Supplementary material for: Reasons for Hospitalizations and Emergency Department Visits Among Patients with Essential Tremor
Source: Tremor Other Hyperkinet Mov (N Y). 2024 Sep 23;14:47. doi: 10.5334/tohm.934 (PMC11428660; doi:10.5334/tohm.934)
Supplement: Supplementary File 1. — Figure 1 and Tables 1 to 4. [file tohm-14-1-934-s1.zip › tohm-934_howard-s1/Supplementary Table 2.docx]

**Supplementary Table 2.** Inpatient admissions associated with each diagnostic category among inpatient admissions of patients with essential tremor (ET) and control patients without ET. Abbreviations: SCI spinal cord injury; TBI traumatic brain injury.

| Principal Diagnostic Category | Control Admissions  (total N = 888)  n (%) | ET Admissions  (total N = 888)  n (%) |
| --- | --- | --- |
| Circulatory | 176 (19.8) | 163 (18.4) |
| Congenital Abnormality | 3 (0.3) | 2 (0.2) |
| Digestive | 122 (13.8) | 93 (10.5) |
| Endocrine | 48 (5.4) | 29 (3.3) |
| Genitourinary | 73 (8.2) | 61 (6.9) |
| Hematologic | 14 (1.6) | 11 (1.2) |
| Infectious Disease | 17 (1.9) | 23 (2.6) |
| Musculoskeletal | 105 (11.8) | 120 (13.5) |
| Neoplasm-related | 86 (9.7) | 69 (7.8) |
| Neurologic | 37 (4.2) | 124 (14.0) |
| Ophthalmologic | 1 (0.1) | 2 (0.2) |
| Psychiatric | 7 (0.8) | 7 (0.8) |
| Reproductive | 9 (1.0) | 9 (1.0) |
| Respiratory | 84 (9.5) | 97 (10.9) |
| Skin and Subcutaneous Tissue | 16 (1.8) | 9 (1.0) |
| Traumatic Injury (other than TBI or SCI) | 20 (2.3) | 15 (1.7) |
| Undefined Organ System | 68 (7.7) | 52 (5.9) |
| Wound-related | 1 (0.1) | 1 (0.1) |
