# Supplementary material for: Reasons for Hospitalizations and Emergency Department Visits Among Patients with Essential Tremor
Source: Tremor Other Hyperkinet Mov (N Y). 2024 Sep 23;14:47. doi: 10.5334/tohm.934 (PMC11428660; doi:10.5334/tohm.934)
Supplement: Supplementary File 1. — Figure 1 and Tables 1 to 4. [file tohm-14-1-934-s1.zip › tohm-934_howard-s1/Supplementary Table 3.docx]

**Supplementary Table 3.** Odds of inpatient admission associated with each diagnostic category among admissions of patients with essential tremor (ET) (n = 419) compared to matched admissions among control patients without ET (n = 867) who had a single admission during the study period. Due to multiple comparisons, statistical significance was defined as a *p*-value of less than 0.002. Abbreviations: CI confidence interval, SCI spinal cord injury, TBI traumatic brain injury.

| Principal Diagnostic Category | Odds Ratio (95% CI) | p-value |
| --- | --- | --- |
| Circulatory | 0.86 (0.64 – 1.16) | 0.33 |
| Congenital Abnormality | 0.69 (0.07 – 6.65) | 0.75 |
| Digestive | 0.40 (0.25 – 0.62) | **<0.001** |
| Endocrine | 0.54 (0.28 – 1.04) | 0.06 |
| Genitourinary | 0.62 (0.38 – 1.00) | 0.05 |
| Hematologic | 0.89 (0.34 – 2.34) | 0.81 |
| Infectious Disease | 1.11 (0.48 – 2.54) | 0.81 |
| Musculoskeletal | 1.42 (1.02 – 1.98) | 0.04 |
| Neoplasm-related | 1.16 (0.79 – 1.70) | 0.44 |
| Neurologic | 6.05 (3.95 – 9.26) | **<0.001** |
| Ophthalmologic | 2.07 (0.13 – 33.26) | 0.61 |
| Psychiatric | 1.18 (0.34 – 4.08) | 0.79 |
| Reproductive | 1.86 (0.75 – 4.60) | 0.18 |
| Respiratory | 0.86 (0.56 – 1.31) | 0.49 |
| Skin and Subcutaneous Tissue | 0.26 (0.06 – 1.03) | 0.05 |
| Traumatic Injury (other than TBI or SCI) | 0.72 (0.30 – 1.73) | 0.46 |
| Undefined Organ System | 0.43 (0.24 – 0.77) | 0.005 |
