# Supplementary material for: Reasons for Hospitalizations and Emergency Department Visits Among Patients with Essential Tremor
Source: Tremor Other Hyperkinet Mov (N Y). 2024 Sep 23;14:47. doi: 10.5334/tohm.934 (PMC11428660; doi:10.5334/tohm.934)
Supplement: Supplementary File 1. — Figure 1 and Tables 1 to 4. [file tohm-14-1-934-s1.zip › tohm-934_howard-s1/Supplementary Table 3.pdf]

Supplementary Table 3. Odds of inpatient admission associated with each diagnostic category among admissions of patients with essential tremor (ET) (n = 419) compared to matched admissions among control patients without ET (n = 867) who had a single admission during the study period. Due to multiple comparisons, statistical significance was defined as a *p*-value of less than 0.002. Abbreviations: CI confidence interval, SCI spinal cord injury, TBI traumatic brain injury.

Principal Diagnostic Category	Odds Ratio (95% CI)	p-value
Circulatory	0.86 (0.64 – 1.16)	0.33
Congenital Abnormality	0.69 (0.07 – 6.65)	0.75
Digestive	0.40 (0.25 – 0.62)	**<0.001**
Endocrine	0.54 (0.28 – 1.04)	0.06
Genitourinary	0.62 (0.38 – 1.00)	0.05
Hematologic	0.89 (0.34 – 2.34)	0.81
Infectious Disease	1.11 (0.48 – 2.54)	0.81
Musculoskeletal	1.42 (1.02 – 1.98)	0.04
Neoplasm-related	1.16 (0.79 – 1.70)	0.44
Neurologic	6.05 (3.95 – 9.26)	**<0.001**
Ophthalmologic	2.07 (0.13 – 33.26)	0.61
Psychiatric	1.18 (0.34 – 4.08)	0.79
Reproductive	1.86 (0.75 – 4.60)	0.18
Respiratory	0.86 (0.56 – 1.31)	0.49
Skin and Subcutaneous Tissue	0.26 (0.06 – 1.03)	0.05
Traumatic Injury (other than TBI or SCI)	0.72 (0.30 – 1.73)	0.46
Undefined Organ System	0.43 (0.24 – 0.77)	0.005
