# Supplementary material for: Reasons for Hospitalizations and Emergency Department Visits Among Patients with Essential Tremor
Source: Tremor Other Hyperkinet Mov (N Y). 2024 Sep 23;14:47. doi: 10.5334/tohm.934 (PMC11428660; doi:10.5334/tohm.934)
Supplement: Supplementary File 1. — Figure 1 and Tables 1 to 4. [file tohm-14-1-934-s1.zip › tohm-934_howard-s1/Supplementary Table 4.docx]

**Supplementary Table 4.** Emergency department (ED) visits associated with each diagnostic category among inpatient admissions of patients with essential tremor (ET) and control patients without ET. Abbreviations: SCI spinal cord injury, TBI traumatic brain injury.

| Diagnostic Category | Control ED Visits  (total N = 1,114)  n (%) | ET ED Visits  (total N = 1,114)  n (%) |
| --- | --- | --- |
| Burn | 1 (0.1) | 1 (0.1) |
| Circulatory | 70 (6.3) | 70 (6.3) |
| Digestive | 166 (14.9) | 136 (12.2) |
| Endocrine | 18 (1.6) | 24 (2.2) |
| Foreign body-related | 4 (0.4) | 3 (0.3) |
| Genitourinary | 96 (8.6) | 74 (6.6) |
| Hematologic | 6 (0.5) | 5 (0.4) |
| Infectious Disease | 23 (2.1) | 24 (2.2) |
| Musculoskeletal | 167 (15.0) | 184 (16.5) |
| Neoplasm-related | 3 (0.3) | 2 (0.2) |
| Neurologic (non-traumatic) | 14 (1.3) | 29 (2.6) |
| Ophthalmologic | 9 (0.8) | 19 (1.7) |
| Otologic | 6 (0.5) | 8 (0.7) |
| Psychiatric | 51 (4.6) | 40 (3.6) |
| Reproductive | 8 (0.7) | 13 (1.1) |
| Respiratory | 71 (6.4) | 102 (9.2) |
| Skin and Subcutaneous Tissue | 29 (2.6) | 30 (2.7) |
| Traumatic Brain or Spinal Cord Injury | 4 (0.4) | 2 (0.2) |
| Traumatic Injury (other than TBI or SCI) | 168 (15.1) | 149 (13.4) |
| Undefined Organ System | 158 (14.2) | 168 (15.1) |
| Wound-related | 33 (3.0) | 31 (2.8) |
