# Supplementary material for: Reasons for Hospitalizations and Emergency Department Visits Among Patients with Essential Tremor
Source: Tremor Other Hyperkinet Mov (N Y). 2024 Sep 23;14:47. doi: 10.5334/tohm.934 (PMC11428660; doi:10.5334/tohm.934)
Supplement: Supplementary File 1. — Figure 1 and Tables 1 to 4. [file tohm-14-1-934-s1.zip › tohm-934_howard-s1/Supplementary Table 4.pdf]

Supplementary Table 4. Emergency department (ED) visits associated with each diagnostic category among inpatient admissions of patients with essential tremor (ET) and control patients without ET. Abbreviations: SCI spinal cord injury, TBI traumatic brain injury.

Diagnostic Category	Control ED Visits (total N = 1,114) n (%)	ET ED Visits (total N = 1,114) n (%)
Burn	1 (0.1)	1 (0.1)
Circulatory	70 (6.3)	70 (6.3)
Digestive	166 (14.9)	136 (12.2)
Endocrine	18 (1.6)	24 (2.2)
Foreign body-related	4 (0.4)	3 (0.3)
Genitourinary	96 (8.6)	74 (6.6)
Hematologic	6 (0.5)	5 (0.4)
Infectious Disease	23 (2.1)	24 (2.2)
Musculoskeletal	167 (15.0)	184 (16.5)
Neoplasm-related	3 (0.3)	2 (0.2)
Neurologic (non-traumatic)	14 (1.3)	29 (2.6)
Ophthalmologic	9 (0.8)	19 (1.7)
Otologic	6 (0.5)	8 (0.7)
Psychiatric	51 (4.6)	40 (3.6)
Reproductive	8 (0.7)	13 (1.1)
Respiratory	71 (6.4)	102 (9.2)
Skin and Subcutaneous Tissue	29 (2.6)	30 (2.7)
Traumatic Brain or Spinal Cord Injury	4 (0.4)	2 (0.2)
Traumatic Injury (other than TBI or SCI)	168 (15.1)	149 (13.4)
Undefined Organ System	158 (14.2)	168 (15.1)
Wound-related	33 (3.0)	31 (2.8)
